# Recent Advances in Metal–Organic Framework (MOF)-Based Composites for Organic Effluent Remediation

**DOI:** 10.3390/ma17112660

**Published:** 2024-06-01

**Authors:** Shuxian Tang, Yuxuan Wang, Peng He, Yan Wang, Gang Wei

**Affiliations:** College of Chemistry and Chemical Engineering, Qingdao University, Qingdao 266071, China; tangshuxian123@outlook.com (S.T.); wangyuxuan831@outlook.com (Y.W.); hepeng_qdu@outlook.com (P.H.)

**Keywords:** two-dimensional materials, metal–organic frameworks, composites, functionalization, organic effluents

## Abstract

Environmental pollution caused by organic effluents emitted by industry has become a worldwide issue and poses a serious threat to the public and the ecosystem. Metal–organic frameworks (MOFs), comprising metal-containing clusters and organic bridging ligands, are porous and crystalline materials, possessing fascinating shape and size-dependent properties such as high surface area, abundant active sites, well-defined crystal morphologies, and huge potential for surface functionalization. To date, numerous well designated MOFs have emerged as critical functional materials to solve the growing challenges associated with water environmental issues. Here we present the recent progress of MOF-based materials and their applications in the treatment of organic effluents. Firstly, several traditional and emerging synthesis strategies for MOF composites are introduced. Then, the structural and functional regulations of MOF composites are presented and analyzed. Finally, typical applications of MOF-based materials in treating organic effluents, including chemical, pharmaceutical, textile, and agricultural wastewaters are summarized. Overall, this review is anticipated to tailor design and regulation of MOF-based functional materials for boosting the performance of organic effluent remediation.

## 1. Introduction

Organic wastewater, typically discharged from industries such as paper, leather, and food processing, contains high concentrations of organic compounds exceeding 2000 mg/L. These pollutants include carbohydrates, fats, proteins, cellulose, and other organic matter, posing a significant risk of pollution if discharged directly into the environment [1,2]. While various methods exist for treating wastewater, including adsorption, flocculation, membrane filtration, electrolysis, oxidation, and biological treatment, they often fall short of completely removing all pollutant activities [3,4,5].

The use of functional materials with high adsorption capacity has garnered considerable attention due to their ease of operation, cost-effectiveness, regulated functions, and ability to be reused [4,6,7,8]. There is, therefore, an urgent need to develop efficient adsorption materials with maximum adsorption capacity and selectivity for toxic substances. While some commercial adsorbents like activated carbon and zeolites have been widely utilized [7,9], they are limited by inherent drawbacks such as amorphous pore structure and fewer active sites.

As a new type of functional nanomaterials, metal–organic frameworks (MOFs) offer intriguing physical and chemical properties that make them highly suitable for various applications [10,11], particularly wastewater treatment and the removal of toxic substances from food and water [10,12]. Unlike traditional adsorbents, MOFs possess unique characteristics such as a large specific surface area, customizable framework structure, tunable pore size, metal unsaturated sites, and the potential to make facile modifications. One notable feature of MOFs is their photo-responsiveness, allowing them to absorb light via their metallic or organic linkages [13]. This property makes MOFs excellent photocatalysts, leveraging their high specific surface area and well-defined structure to enhance the translocation of target molecules. As a result, MOFs hold great promise for addressing environmental pollution and advancing sustainable water-treatment technologies [14].

MOFs can exhibit instability and susceptibility to decomposition when exposed to hydrophilic environments. To address this challenge, MOFs are often integrated with functional or matrix materials to create novel composites with improved flexibility and water stability [12,15,16]. These composite materials, such as MOF-based hydrogels and aerogels and their derivatives, have diverse applications in treating organic wastewater, including drug residues and dyes [17,18]. By leveraging the tunable properties of MOFs and their compatibility with selective layers, MOF membranes offer adjustable internal structures and demonstrate versatility in organic wastewater treatment. Various nano-MOF materials have been incorporated into conventional polymer membrane structures to impart desirable properties such as high water-flux and enhanced selectivity [19,20,21]. Additionally, the integration of nano-MOFs enhances membrane durability, ensuring the production of long-lasting membranes for efficient wastewater treatment processes.

It is impressive to see the substantial progress made in utilizing MOF-based functional materials for environmental science and water purification. Several important reviews have contributed significantly to our understanding of MOFs’ potential in removing organic contaminants from water. For instance, Rojas et al. provided a comprehensive review on synthesizing MOFs for removing various organic contaminants from water [22]. Their focus on the adsorption and degradation of MOFs in emerging and known organic pollutants provides insights for effective water remediation strategies. Drout and colleagues presented a review on designing and synthesizing Zr-MOFs specifically for adsorbing organic pollutants [23]. Their emphasis on understanding the structure–property relationships of Zr-MOFs enriches our knowledge of MOF-based adsorption mechanisms. Tchinsa and colleagues highlighted the design and construction of MOF-based adsorbents tailored for removing organic pollutants from aqueous solutions [24]. Their analysis of MOF adsorbents’ performance and strategies for synthesizing cost-effective MOF materials offer valuable insights for practical water treatment applications.

**Scheme 1:**
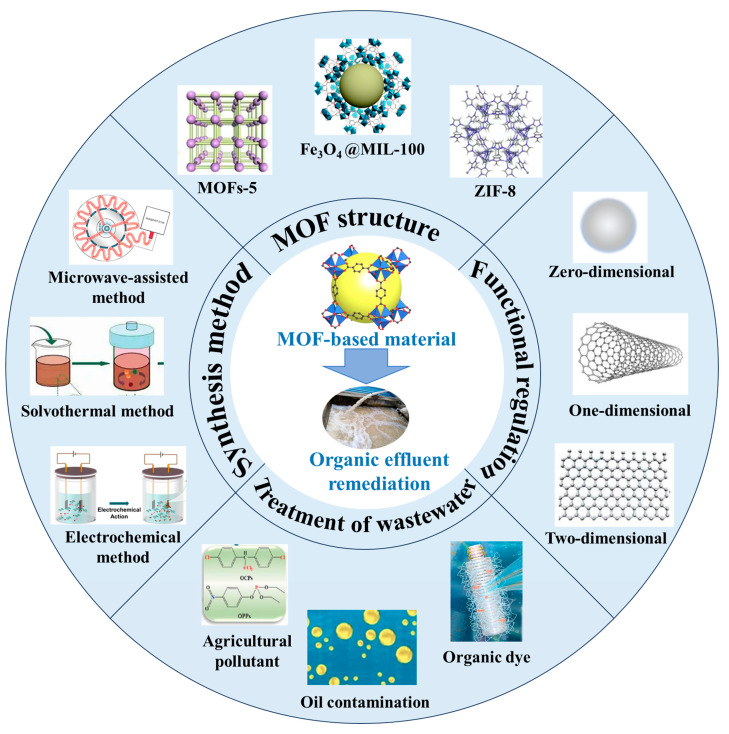
Schematic presentation of the synthesis, functionalization, and organic effluent remediation of MOF-based composite materials.

After examining these previously published reviews, we realized that MOFs could still be further investigated in the treatment of organic wastewater, especially in modulating the structure and function of MOFs to remediate organic pollutants more effectively. Therefore, in this review, we demonstrate recent advances in the regulation of MOFs for the treatment of organic effluents (Scheme 1). We begin by discussing the synthesis methods of MOF-based materials, focusing on conventional and electrochemical methods. Subsequently, we summarize the structural and functional modulation of MOF-based materials. Finally, we delve into their applications in organic wastewater treatment. We believe that this work could provide additional insights into the preparation of cost-effective MOF-based adsorbents for high-performance water purification.

## 2. Synthesis Methods of MOF-Based Materials

**Figure 1:**
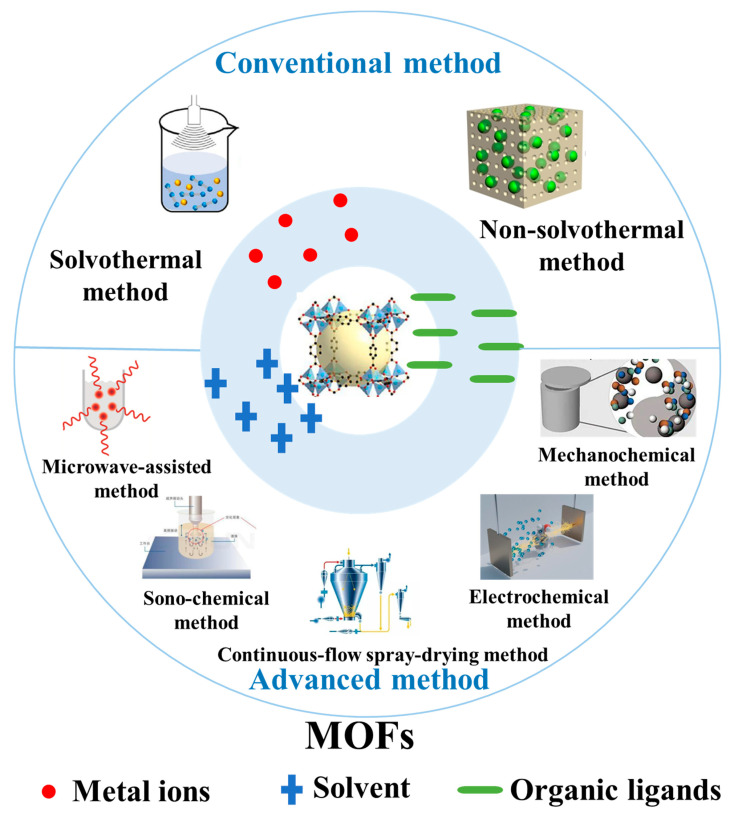
Typical synthesis methods of MOFs and MOF-based materials, which include conventional and advanced methods.

Since the discovery of MOFs, various synthesis methods have been developed, including sonochemistry, electrochemistry, solvothermal, and microwave-assisted methods. Purification and activation are two crucial steps following the synthesis of MOFs [25]. Impurities in the MOF can diminish its adsorption capacity and properties, making activation necessary through heating, which can sometimes result in the collapse of the MOF network and guest molecules. To simplify the activation process, different methods can be employed, such as replacing non-volatile solvents with volatile ones to reduce the required deactivation temperature. In this section, we introduce two traditional and advanced methods of MOF synthesis, as depicted in Figure 1.

### 2.1. Conventional Methods for the Synthesis of MOFs

Traditional methods for the synthesis of MOFs include the solvothermal and non-solvothermal techniques. In this section, we will present some details on both types of synthesis protocols.

**Figure 2:**
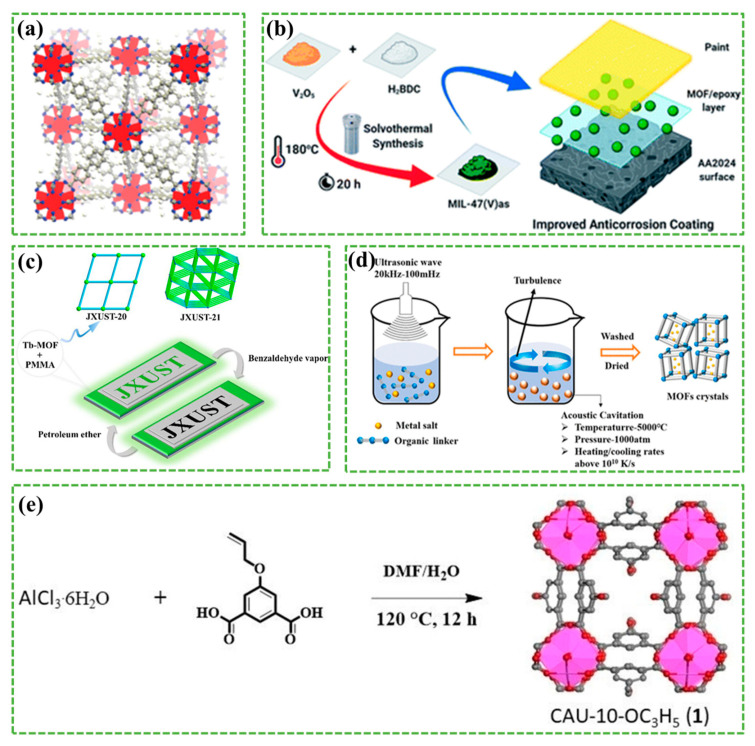
Traditional synthesis methods of MOFs: (**a**) UiO-67 with BPDC as a linker. Reprinted with permission from [28]. (**b**) Synthesis of vanadium MIL-47 MOF. Reprinted with permission from [31]. (**c**) Synthesis of MOFs based on H_2_BTDC under solvothermal conditions. Reprinted with permission from [32]. (**d**) Solvothermal synthesis of MOFs containing transition metals. Reprinted with permission from [33]. (**e**) Synthesis of CAU-10-OC_3_H_5_ MOF. Reprinted with permission from [34].

The solvothermal method involves synthesizing MOFs by conducting reactions at temperatures above the boiling point of the solvent in a specialized closed chemical reactor under high pressure generated by solvent vapor or a pump [26]. Due to the high temperature and pressure conditions, the organic ligand exhibits better solubility in the solvent, and the elevated temperature allows for slow crystallization, resulting in improved crystal morphology. Consequently, the solvothermal method typically yields higher product yields and better crystallinity [27]. Common MOFs synthesized via the solvothermal method include UiO-67 (Figure 2a), ZIF-9, MIL-74, and others [28,29,30].

In solvothermal methods, MOFs are synthesized in sealed nuclear magnetic resonance (NMR) tubes or small vials using conventional electric heating in controlled intervals [35]. For instance, Huang et al. utilized this approach to fabricate Cu_3_(benzene tricarboxylic acid)_2_ (Cu_3_(BTC)_2_) and Cu (benzene dicarboxylic acid) (Cu(BDC)) MOFs for treating phenol wastewater [36]. Similarly, Chen et al. successfully developed a heterogeneous MOF-In/Gd cyclobutane-1,2,3,4-teracarboxylicdianhydride(CBDA) using a “one-pot” solvothermal method with a single functional connector CBDA [37]. Their research laid the groundwork for the advancement of heterogeneous metal MOF materials and facilitated the progress of functional MOF materials.

Liang et al. synthesized Mn-MOF cluster structures using the solvothermal method, demonstrating its efficacy as a highly active air electrode catalyst for lithium–oxygen batteries [38]. The air electrode incorporating this MOF material exhibited improved reversibility and oxidation processes. In a separate study, Kim and colleagues employed the solvothermal method to synthesize three-column layered MOFs based on M(HBTC) (4,4′-bipy)·3DMF (M=Ni, Co, and Zn), which were utilized as novel catalysts for solvent-free CO_2_ epoxide cycloaddition reactions [39]. Wang et al. synthesized two novel lanthanide-MOFs based on 2,1,3-benzothiadiazo-4,7-dicarboxylic acid (H_2_BTDC) under solvothermal conditions [32], as shown in Figure 2c. These MOFs provided a simple and efficient platform for detecting volatile organic compounds (VOCs) in environmental science.

Zhang et al. developed a simple and template-free solvothermal method as a bottom-up approach to synthesize mesoporous/macroporous MOF nanosheets in a simple and scalable manner [40]. Zorainy et al. successfully synthesized vanadium MIL-47 metal-organic skeletons using the solvothermal method [31], as indicated in Figure 2b. Compared with MOFs obtained by other synthesis techniques, the synthesized MOFs exhibited similar thermal stability.

Cation exchange polymer resin beads have been utilized as heterogeneous controlled release sources of metal cations for the solvothermal synthesis of new transition metal–organic frameworks with phase purity and/or high yields. The introduction of resin beads has significantly affected the results of the solvothermal reaction. Du et al. proposed a new resin-assisted solvothermal synthesis method for a series of novel MOFs (Fe/Co/Ni/Cu MOFs) [33]. This method offered a new possibility for the preparation of novel MOFs, as well as a more efficient way to prepare pure and more crystalline known and unknown phases (Figure 2d). Ghosh et al. introduced a new MOF called Al-CAU-10-OC_3_H_5_, prepared by incorporating allyl-functionalized 5-(allyl) isophthalic acid (H_2_AIA) as a connector via a traditional solvothermal process [34], as presented in Figure 2e.

While the solvothermal method offers numerous advantages, strict regulation of time and temperature is essential. Temperature variations can impact particle morphology, and extended reaction times may result in the degradation of MOFs [41].

Non-solvothermal synthesis occurs at room temperature in an open system, at temperatures below the boiling point of the solvent. Unlike solvothermal methods, non-solvothermal methods do not necessitate specialized complex reaction vessels and can be conducted at room temperature or with mild heating conditions [42]. Common MOFs such as MOF-5, MOF-74, ZIF-8, and MOF-177 have been synthesized using non-solvothermal methods [43,44,45,46].

Non-solvothermal methods offer an easier route to synthesizing MOFs compared to solvothermal methods. Mechanical, nano-precipitation, and emulsion methods are non-solvothermal techniques for the rapid growth of MOFs. In this approach, complex equipment is not required, and MOFs can be produced in open containers at the boiling temperature and atmospheric pressure of the solvent. The adjustment of pH value and temperature is crucial for achieving the maximum yield of MOF material. For example, Zhang et al. employed an adaptive method that omitted microwave/ultrasonic treatment and additional stress [46]. The solution of Zn(OAc)_2_·2H_2_O and 2,5-dihydroxyterephthalic acid were dissolved in dimethylformamide and then added to the salt solution and stirred at room temperature for 18 h to prepare MOF-74 (Zn). Finally, MOF-74 (Zn) was added to an ethylene–diamine–toluene solution and dried in air to obtain ammoniated MOF-74 (Zn), which served as a light-emitting sensor for the selective detection of tetrabrombisphenol A.

Compared with traditional adsorbents, MOF-based materials have high specific surface area, abundant active sites, high porosity, and good catalytic activity, as well as the disadvantage of being expensive. To address this cost issue, some researchers have proposed combining expensive MOF-based materials with ubiquitous, low-cost, and durable materials to form novel MOF composites. The composites resulting from this combination can minimize and ultimately resolve the cost, durability, and processability issues of MOFs while retaining their valuable structural and chemical properties [47]. Hou et al. combined MIL-53 with a glass matrix through a strategy of heat treatment and ball milling, which preserved the coordination bonding and chemical structure of the MIL-53, resulting in composites with stabilized macroporous conformation. In addition, due to the presence of nanoscale interfacial interactions, the mechanical properties of the composites were improved compared with the initial materials [48], and the composites were promising for membrane separation, gas adsorption and photocatalytic degradation of pollutants [49].

However, traditional methods for synthesizing MOFs typically yield fine powders, which limit their commercial application.

### 2.2. Advanced Methods for the Synthesis of MOFs

#### 2.2.1. Electrochemical Synthesis

The key feature of the electrochemical synthesis method is that the metal ions necessary for forming MOFs are generated during the electrochemical process, rather than being introduced from corresponding salt solutions or produced in reactions between metals and acids. These metal ions are created by etching the anode in an electrolyte solution containing organic ligands and electrolytes, thus circumventing the need for anions to form in a continuous reaction, which is also essential for large-scale MOF production [42]. The electrochemical synthesis method enables the synthesis of MOFs at room temperature and atmospheric pressure, with relatively short reaction times (ranging from tens of minutes to several hours).

**Figure 3:**
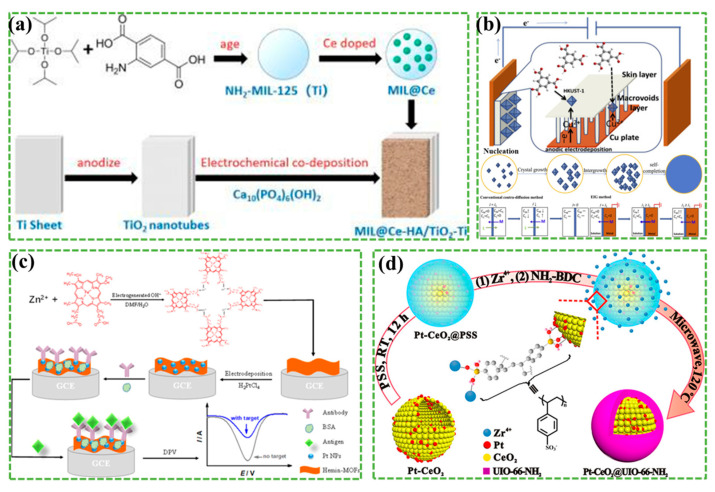
Advanced synthesis methods for MOFs: (**a**) Electrochemical synthesis of TiO_2_, HA, and Ce-doped MIL. Reprinted with permission from [50]. (**b**) Electrochemistry-assisted interface growth of MOFs. Reprinted with permission from [53]. (**c**) Electrosynthesis of hemin-MOFs for electrochemical sensors. Reprinted with permission from [54]. (**d**) Microwave-assisted synthesis of Pt-CeO_2_@UIO-66-NH_2._ Reprinted with permission from [55].

Zhang et al. synthesized novel La-doped ZIF-8 MOF nanoparticles by electrochemical anodic oxidation and electrochemical deposition and used them to optimize hydroxyapatite–titanium dioxide composite coatings (Figure 3a) [50,51]. In their results, they showed that the composite coating could improve the corrosion-resistant and antimicrobial properties of TiO_2_ without affecting its original biocompatibility. Tang et al. established a sensitive, efficient, and label-free electrochemical immune sensor (Figure 3c), by growing 2D hemin-MOFs or 2D/3D hemin-BTC-MOF hybrid nanocomposites (NCs) directly onto the electrode surface through electrosynthesis. The strategy provided an alternative path for designing new functionalized MOF materials for applications in other electrochemical-related fields. Zhan et al. directly prepared polyaniline-@Cu-1,3,5-phenyltriformic acid (PAN@Cu-BTC) NCs with excellent electrochemical activity on the surface of screen-printed electrodes using a two-step electrochemical synthesis method [52]. Then, the tetracycline aptamer was assembled on the electrode surface through the coordination between 5′-PO43- on the aptamer and Cu^2+^ on MOF, and a new type of tetracycline aptamer electrochemical sensor was realized. The aptamer sensor is suitable for the detection of TC residues in fresh aquatic products.

Moreover, environmental applications necessitate dense and immobilized MOF films for sensing and adsorption, which can be precisely fabricated using electrochemical synthesis methods. Zhang et al. employed a novel in situ growth method to synthesize a continuous MOF atop the membrane through electrochemistry-assisted interface growth (Figure 3b), and the resulting Cu-BTC/polyether sulfone membrane exhibited excellent performance in removing dyes from water [53]. This membrane preparation strategy integrates anode dissolution of metals with reverse diffusion, overcoming the limitations of traditional reverse diffusion and interfacial polymerization methods, and bridging the gap between the two approaches. Furthermore, MOF synthesis can be accurately controlled and monitored in real time using this method. This innovative strategy is expected to significantly expand the range of interfacial synthesis of MOFs on porous substrate surfaces for various applications.

Among various methods for synthesizing MOFs, electrochemical synthesis has garnered considerable attention due to its environmentally friendly advantages, such as mild preparation conditions and easy controllability, thereby improving the feasibility of continuous large-scale production of MOFs.

#### 2.2.2. Microwave-Assisted Synthesis

Microwave-assisted synthesis is a rapid and efficient method for preparing MOFs. This approach can yield MOFs with high specific surface area and porosity in a short duration, while allowing for control over the morphology and properties of the products by adjusting the reaction conditions.

The principle underlying microwave synthesis of MOFs involves utilizing microwave energy to expedite the reaction rate, thereby facilitating the completion of the synthesis reaction within a brief period. During this process, metal ions and organic ligands rapidly react to form MOFs under the influence of microwave fields. In comparison to traditional thermal synthesis methods, microwave synthesis of MOFs offers several advantages: (i) Fast reaction rate: microwaves can accelerate the reaction rate in a short time, so as to achieve rapid synthesis. (ii) Uniform product quality: microwaves can evenly heat the reaction system to avoid the problem of uneven product quality. (iii) High product purity: due to the fast reaction rate, the microwave method can complete the reaction in a short time, thereby reducing the impurities in the product. (iv) Controlled product morphology: The reaction conditions of microwave synthesis of MOFs can be controlled by adjusting the microwave power, reaction time, and reactant proportion. Indeed, microwave synthesis of MOFs has emerged as a widely employed method, capable of producing various MOFs with specific morphologies and properties to address the diverse application requirements across different fields.

Long et al. developed a microwave-assisted technique for the controlled synthesis of Pt/CeO_2_@MOF core@shell hybrids [55]. As shown in Figure 3d, by employing PSS as a modifier and utilizing microwave assistance, MOFs could be continuously grown on Pt/CeO_2_ nanospheres. The resulting Pt-CeO_2_@UIO-66-NH_2_ exhibited high conversion (99.3%) and selectivity (>99%) for furfural selective hydrogenation to furfuryl alcohol. With a similar technique, Li and colleagues proposed a microwave-assisted method for preparing MIL-53 (Fe) octahedrons with uniform concave surfaces [56]. This unique structural geometry allowed MIL-53 (Fe) to capture upconversion nanoparticles (UCNPs) on its concave surface in a stable form. The developed MIL-53 (Fe)/UCNP composites exhibited remarkable photocatalytic activity under near-infrared light.

## 3. Functional Regulations of MOF-Based Materials

### 3.1. Structural Regulation

#### 3.1.1. Aerogels and Hydrogels

When MOFs come into contact with hydrophilic media, their structures tend to become unstable and prone to decomposition. Therefore, MOFs are typically combined with functional or base materials to form novel composite materials with higher flexibility and water stability [57]. Notably, aerogels are solids with large specific surface areas, a basic characteristic of highly porous materials. Additionally, aerogels themselves are metastable [58], making them suitable for various applications such as thermal insulation, gas adsorption, water treatment, and catalysis. Hydrogels, on the other hand, are three-dimensional network structures formed by the physical or chemical crosslinking of polymer monomers, and they have been proven to be effective adsorbents [59]. With their unique 3D structure and abundant functional groups, hydrogel networks offer numerous adsorption sites for binding target molecules. Importantly, hydrogels can absorb water without losing their structural integrity, facilitating the separation and recycling of hydrogel adsorbents from treated wastewater [60]. Therefore, in terms of pore size, porosity, pore volume, and structural stability, MOF and aerogel composite materials offer more advantages than either of the materials alone. Moreover, these mixed materials often exhibit adjustable mechanical toughness.

The structural diversity, stability, structural properties, and application prospects of MOF aerogels/hydrogels are determined by the hierarchical porous tunable structure of the synthesis and drying methods. Hydrogels are primarily prepared by incorporating MOFs into the polymer precursor solution, employing methods such as in situ growth, direct mixing, or chemical cross-linking of the polymer components around the MOFs through free radical polymerization. For the MOF aerogels, a final step involving supercritical CO_2_ drying or freeze-drying is necessary to achieve MOF aerogels with the desired structure and porosity [58].

**Figure 4:**
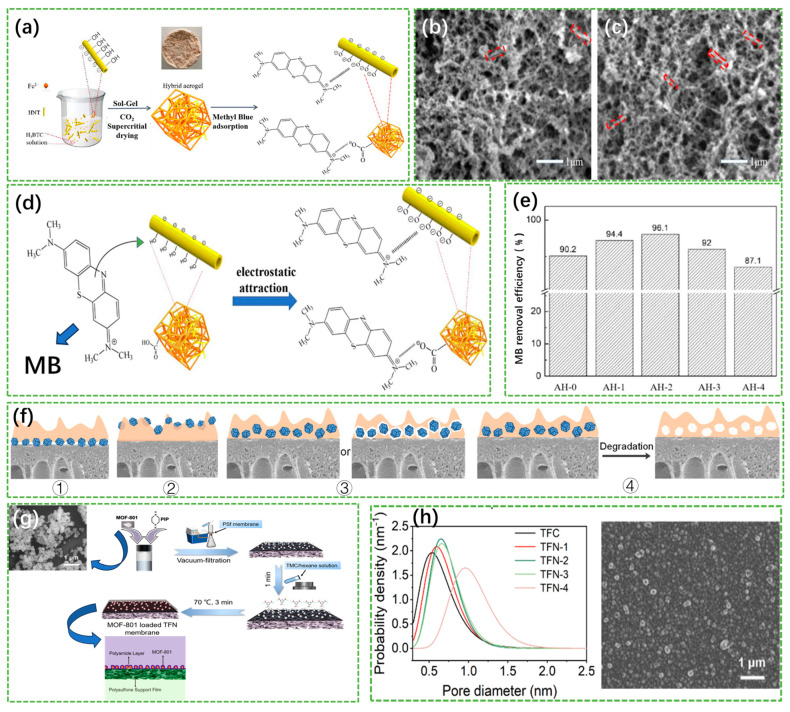
Structural regulation of MOF-based materials: (**a**) Preparation of MOF(Fe)/HNTs composite aerogel and (**b**,**c**) SEM images of aerogels with different 3D porous network structure, (**d**) removal efficiency of MB and RhB by different MOF(Fe)/HNTs composite aerogels, and (**e**) adsorption mechanism of MOF(Fe)/HNTs composite aerogels. Reprinted with permission from [61]. (**f**) MOF-based TFN membranes with different MOF positions and interfaces. Reprinted with permission from [63]. (**g**) Synthesis of MOF-TFN membranes, and (**h**) pore size distribution and SEM image of MOF-TFN membranes. Reproduced with permission from [64].

For instance, Liu et al. prepared MOF(Fe)/halloysite nanotubes (HNTs) composite aerogels using the sol–gel method and supercritical CO_2_ drying (Figure 4a) [61]. By controlling the addition amount of HNTs, the microscopic morphology and pore structure of the aerogel can be properly regulated. The appropriate addition of HNTs helps maintain the network structure, whereas an excessive amount of HNTs tend to cluster together, leading to the collapse of the pore structure and a reduction in the specific surface area (Figure 4b,c). Furthermore, these HNTs are tightly bound to the matrix, resulting in strong interfacial bonds (Figure 4d). Additionally, the incorporation of HNTs increases the number of active sites in the MOF(Fe) aerogel, rendering it a promising adsorbent for water treatment. The MOF(Fe)/HNTs composite aerogel exhibited excellent adsorption performance for MB, with a maximum adsorption capacity of up to 384 mg/g and an adsorption efficiency of up to 96.1% (Figure 4e). It has also been reported that Zhu et al. have successfully prepared tricyclic antidepressants(TCAs) from four different MOF-alginate composites, including HKUST-1, ZIF-8, MIL-100(Fe), and ZIF-67-alginates [62], by using alginate hydrogels crosslinked with different metal ions. The resulting alginate hydrogel/MOF composites were experimentally demonstrated to have a stronger adsorption capacity than the pure hydrogel, making them effective and convenient absorbents for water purification.

Based on the favorable properties of MOF aerogels and hydrogels, these materials are commonly employed in the treatment of organic wastewater. Researchers have demonstrated that MOF aerogels composed of ZIF-8 MOF exhibit rapid adsorption of RhB [65]. This is attributed to the higher proportion of ZIF-8, which results in tighter stacking between MOFs in the MOF aerogel, providing a smaller surface area for larger dyes. The efficacy of the photocatalytic degradation process relies on the ability of the photocatalyst to generate stable electron–hole pairs that produce free radicals (e.g., -OH) for degrading organic dyes. Furthermore, Yaghi’s group reported the preparation of stabilized ZIF-8 MOF in a nitrogen atmosphere at 550 °C, which had a structural stability of 7 days in boiling water. The prepared ZIF-8 also maintained the same crystallinity and porosity with good chemical structural stability in different organic solvents and water [66].

The aforementioned studies highlight how the distinctive pore structure and stability of MOF aerogels and hydrogels contribute to the advancement of reusable materials for water treatment. The microporous nature of MOFs, coupled with the variety of ligands and metal ions, empowers them to efficiently adsorb various harmful substances present in water.

#### 3.1.2. Nanostructured Membranes

MOFs have demonstrated significant potential in enhancing the separation efficiency of thin-film nanocomposite (TFN) membranes owing to the high porosity, low density, large specific surface area, tunable pore properties, and unique topology of MOF materials.

In the preparation of TFNs, the positioning of the MOF materials significantly influences the permeability properties of the nanomembranes, as illustrated in Figure 4f, which depicts the initial case of preloading the MOFs onto the substrate before interfacial polymerization, which reduces membrane crosslinking, resulting in a more porous and permeable selective layer [63]. After this, MOF NPs are dispersed on the upper surface of the membrane, allowing them to directly modify the membrane surface [67]. Then, the presence of channels surrounding the MOFs enhances the porosity of the selective layer, and facilitates water transport. Previously, researchers have utilized MOFs as sacrificial nanofillers to create nanocavities with this technique [68,69], where ZIF-67 MOFs have been employed as self-sacrificial templates to construct TFNs. These resultant membranes demonstrated a removal efficiency of up to 99.2% for Congo red (CR) with a concentration of 500 ppm.

MOF-based TFN membranes have extensive application in organic wastewater treatment owing to their adjustable internal structure. Dai et al. fabricated TFN NF membranes doped with MIL-101 (Cr) [70]. They modified MIL-101 (Cr) with ethylenediamine to create doubly charged MOFs, which were incorporated into the selective layer. This modification enabled the membranes to exhibit enhanced selectivity and water permeability, facilitating the removal of drugs from wastewater. Furthermore, some researchers opted for nano-MOF materials characterized by stability in acidic, alkaline, and aqueous environments, along with large specific surface areas and abundant microporous structures, to construct TFN membranes [71].

Wang and colleagues prepared TFN membranes by depositing MOF-801 NPs onto polysulfone membrane supports using vacuum-assisted filtration, followed by construction through interfacial polymerization [64]. They observed that the incorporation of MOF-801 NPs into the polyamide (PA) layer led to a notable increase in the membrane’s roughness, thereby expanding the effective area available for water transport. Moreover, the hydrophilicity of the membrane was enhanced, the pore size increased, and the PA layer became thinner, all of which favored the improvement of the material’s permeability and osmotic properties, facilitating the accelerated transport of water molecules (see Figure 4g,h). Furthermore, other researchers have combined MOF-based materials with catalytic properties in TFN membranes to develop nanomembranes with high catalytic activity for the photocatalytic degradation of organic pollutants. Bai et al. [72] covalently attached catalyst particles Ag@UiO-66-NH_2_ onto polyamide TFN membranes through interfacial polymerization reactions [73], producing Ag@UiO-66-NH_2_ TFN membranes with high catalytic performance, excellent catalytic cycling capability, and membrane stability for sustained catalytic degradation of RhB.

In addition, we have focused on studies related to the swelling of MOF materials. MIL-88 A is a three-dimensional framework of trimeric or octahedral iron(II) linked to fumaric acid dianions with a very large volume that swells when exposed to polar solvents [74]. The total crystal volume of the MIL-88A crystals increases by about 45% overall at 90% relative humidity. When the relative humidity is reduced to 20%, the crystals return to their initial size, thus exhibiting a reversible expansion/contraction process. The reason for the swelling is as follows: hydrogen bonding interactions are formed between the guest molecules and the backbone, and the unit cell volume of the MOF exhibits a reversible enlargement when polar solvent molecules are adsorbed. In these MOFs, solvation can be strongly influenced by the solvent and the functional groups in the linker groups. In addition to changes at the molecular level, a significant effect of this lattice swelling is the subsequent macroscopic deformation of the crystals. Based on this, some researchers have reported the synthesis of AMIL-88A@PVDF composite membranes with self-folding properties by adding flexible MIL-88A crystals to a PVDF polymer matrix [75]. The composite membrane exhibited a good oil removal rate (98.3%) and high permeation flux (667 L·m^−2^·h^−1^), which was about seven times that of the pure PVDF membrane, indicating that the surface-modified membrane had excellent permeability and good separation performance. Even after four filtration cycles of the oil–water emulsion, the retention rate remained above 97%, showing good reusability [76]. In summary, various nano-MOF materials have been incorporated into the structures or surfaces of conventional polymer films to enhance the adsorption and photocatalytic properties of MOF-based materials. This is achieved by regulating parameters such as porosity, surface area, pore size, and host–guest interactions, resulting in improved water flux, selectivity, and fouling resistance. Furthermore, integrating nano-MOFs into the membrane structure enhances durability and ensures the production of long-lasting membranes.

### 3.2. Functional Regulation

MOF-based materials possess unique properties such as excellent porosity, tunable structure, uniform component dispersion, and controllable pore size. However, they also suffer from certain drawbacks, including susceptibility to rapid hydrolysis, lack of stability, and poor electrical conductivity [77]. Combining MOF-based materials with different substances through compositing is an effective approach to enhance their performance and efficiency. Consequently, some researchers have endeavored to leverage their unique pore structure to accommodate additional guest materials with outstanding properties, thereby achieving more comprehensive catalytic effects [78], adsorption properties, and stability compared to single-component systems, owing to the synergistic interactions between the guest-substrate MOF materials. In the following sections, we will discuss the compositing of 0D, 1D, and 2D nanomaterials with MOFs to modulate their performance.

#### 3.2.1. Composites of 0D Materials with MOF-Based Materials

The composites of metal nanoparticles (MNPs) and MOFs combine the advantages of both components while compensating for their individual shortcomings. Incorporating MNPs addresses the deficiency in electrical conductivity often found in MOFs. Additionally, the synergy between MOFs and MNPs helps prevent particle agglomeration and enables effective control over NP growth [79].

**Figure 5:**
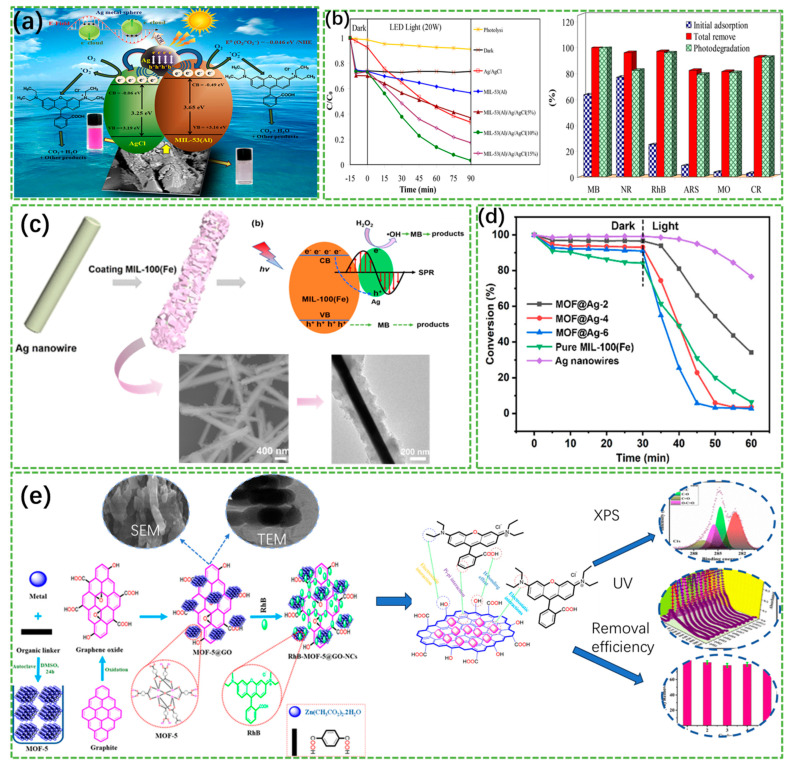
Functional regulations of MOF-based materials: (**a**) Addition of MNPs for the synthesis of MIL-53(Al)/Ag/AgCl NCs and (**b**) degradation and adsorption of RhB by MIL-53(Al)/Ag/AgCl NCs. Reprinted with permission from **[66]**. (**c**) Preparation of MOF-AgNW NCs for (**d**) photocatalytic degradation of MB. Reproduced with permission from [80]. (**e**) Preparation of MOF-5@GO NCs for the adsorption of RhB. Reproduced with permission from [81].

The noble MNPs embedded in MOFs exhibit high activity and good selectivity in multiphase catalytic reactions due to the synergistic effect between these two functional materials, making them versatile photocatalysts. Khodayari et al. prepared MIL-53(Al)/Ag/AgCl NCs by a simple reflux method [66], which served as a novel plasma photocatalyst and exhibited the removal ability of the composite for three cationic dyes, Nile red(NR), MB and RhB (Figure 5a). They conducted photocatalytic experiments under LED light irradiation and examined the impact of NCs’ mass ratio on the photocatalytic performance of MIL-53(Al)/Ag/AgCl. The findings confirmed that the interaction between MIL-53(Al)/Ag/AgCl NPs and MIL-53(Al) contributed to the excellent photocatalytic capability of MIL-53(Al)/Ag/AgCl NC. The notable surface area of MIL-53(Al)/Ag/AgCl NC, coupled with the presence of benzene rings in the structure of MIL-53(Al), offered ample opportunities for dye molecule adsorption, thereby providing more active sites for dye adsorption and enhancing the photocatalytic activity. Among the tested compositions, MIL-53(Al)/Ag/AgCl (10%) demonstrated the highest catalytic efficiency for the photodegradation of various dye pollutants (Figure 5b).

#### 3.2.2. Composites of 1D Materials with MOF-Based Materials

In recent years, several silver-modified MOF composites have demonstrated effective photocatalytic degradation of organic dyes [82,83]. However, most of these reports have focused on incorporating AgNPs into MOF-based materials, with limited exploration of silver nanowires (AgNWs). Research on the controlled combination of AgNWs and MOFs, such as the uniform coating of a MOF layer onto AgNWs, and the investigation of synergistic effects between them, holds significant promise for applications in photocatalytic degradation of organic dyes.

Chen et al. combined the surface of MIL-100 (Fe), an iron-based MOF assembled from Fe^3+^ ions coordinated with tricarboxylic acid [80], with plasma resonance-excited AgNWs to photocatalytically degrade MB, as investigated in photocatalytic experiments (Figure 5c). The results demonstrated that the photogenerated electron–hole separation efficiency of the MOF-AgNW hybrid catalysts was enhanced. The thicker MOF coating provided a larger BET surface area, thereby improving the photocatalytic activity while maintaining optimal adsorption capacity and adsorption rate (Figure 5d). In addition, the researchers operated MoF@Ag-6 for five cycles and repeated degradation performance and observed no significant loss of catalytic activity and crystallinity of MoF@Ag-6, suggesting that the composite catalyst is stable. Additionally, some researchers have developed molecularly imprinted sensors based on Co_3_O_4_ nanowires and Co_3_O_4_@MOF-74 NCs [84], which exhibited high selectivity, stability, and reproducibility in detecting the organophosphorus insecticide cyanamidophos. These sensors leveraged the high sensitivity characteristics and specific surface area of Co_3_O_4_ nanowires, along with the synergistic effect between Co_3_O_4_ nanowires and MOF-74, which facilitated fast electron-transfer rates, resulting in the sensor’s high selectivity and sensitivity for detecting organophosphorus insecticides.

#### 3.2.3. Composites of 2D Materials with MOF-Based Materials

The integration of graphene with MOF materials offers numerous advantages. Graphene’s 2D honeycomb carbon structure provides exceptional stability and high electrical conductivity. When combined with MOFs in a controlled manner, graphene can enhance the stability, adsorption capacity, and photoelectrocatalytic properties of MOFs. This synergistic combination leverages the unique properties of both materials to create composite materials with enhanced performance for various applications.

Kumar et al. combined graphene oxide (GO) sheets with MOF materials using a stepwise synthesis method to prepare MOF-5@GO NCs [81], featuring a mesoporous structure with a relatively high specific surface area of 135.37 m^2^/g and an average pore size of 0.014 nm (Figure 5e). The presence of unsaturated bonds (C=C and C=O) in MOF-5 results in a negatively charged graphene surface, while RhB is positively charged, facilitating ionic interactions between RhB and MOF-5@GO NCs. Additionally, RhB contains C=C double bonds and π-electrons, which can readily interact with the π-electrons on the benzene rings present on the surface of MOF-5 and graphene through π-π interactions. Moreover, the composite exhibited high porosity, further enhancing its ability to effectively adsorb RhB. Meanwhile, the researchers determined the regeneration ability of the MOF-5@GO nanocomposites, and it was observed that the adsorption rate of the MOF-5@GO nanocomposites showed an insignificant decrease after the first three cycles but remained unchanged in the last two cycles. These results indicate that the MOF-5@GO nanocomposites have fast adsorption integration rates and good reversibility.

The integration of GO with MOFs has been explored by Jabbari and colleagues, who prepared Fe_3_O_4_/Cu-BTC@GO composites using an in situ growth method for the adsorptive removal of MB from aqueous solutions [85]. The presence of active functional groups such as carboxyl and hydroxyl groups on the surface of GO increased the active sites in the graphene/MOF composites, enhancing electrostatic attraction between the MOFs and organic dyes or pesticides. Additionally, the formation of pores between the GO substrate and the MOFs contributed to a significant enhancement in the physical adsorption capacity. The unsaturated bonds and charge interactions between -COOH and MB on the GO surface further increased the adsorption capacity, resulting in excellent performance in removing MB. Similarly, Zhao et al. synthesized SnO_2_@UiO-66/rGO composites using a stepwise synthesis method [86]. These composites exhibited enhanced photoreactivity and photocatalytic properties attributed to effective charge transfer, a well-defined microporous structure, improved visible light absorption, large specific surface area, and synergistic interactions between MOFs and graphene. These factors led to a significant increase in the catalytic degradation rate of RhB under visible light.

In summary, composites featuring graphene with a 2D structure can enhance the stability, conductivity, and catalytic activity of MOFs in aqueous solutions. This is due to the abundance of reactive groups such as -COOH and -OH on GO sheets, which provide binding sites for MOFs, effectively immobilizing MOF nanoparticles on the GO sheets [87,88]. Moreover, the π-π stacking between graphene sheets enhanced the interaction and binding of MOFs with graphene [89]. Therefore, the composite of GO with MOFs materials improved the charge transfer efficiency [90] and the stability of MOFs materials in the aqueous phase [91], and also enhanced the adsorption performance of organic pollutants.

## 4. Application of MOF-Based Materials for Treating Organic Effluents

In recent decades, rapid urbanization and population growth have led to the discharge of significant amounts of organic pollutants into the environment [92,93]. Organic wastewater, typically originating from industries such as paper, leather, and food processing, often contains high concentrations of carbohydrates, fats, proteins, cellulose, and other organic compounds. Direct discharge of such wastewater can result in severe pollution. While various methods exist for removing contaminants from wastewater, such as adsorption, flocculation, membrane filtration, electrolysis, oxidation, and biological treatment, none are universally effective in eliminating all pollutant activity [94,95].

MOFs hold great promise for treating wastewater and removing toxic substances from food and water. Their key attributes include a large specific surface area, customizable framework structure, tunable pore size, metal unsaturated sites, and ease of modification. Moreover, MOFs exhibit photoresponsive properties, capable of light absorption through their metal or organic linkages. With their high specific surface area and well-defined structure, MOFs serve as excellent photocatalysts, facilitating the translocation of target molecules [96,97].

**Figure 6:**
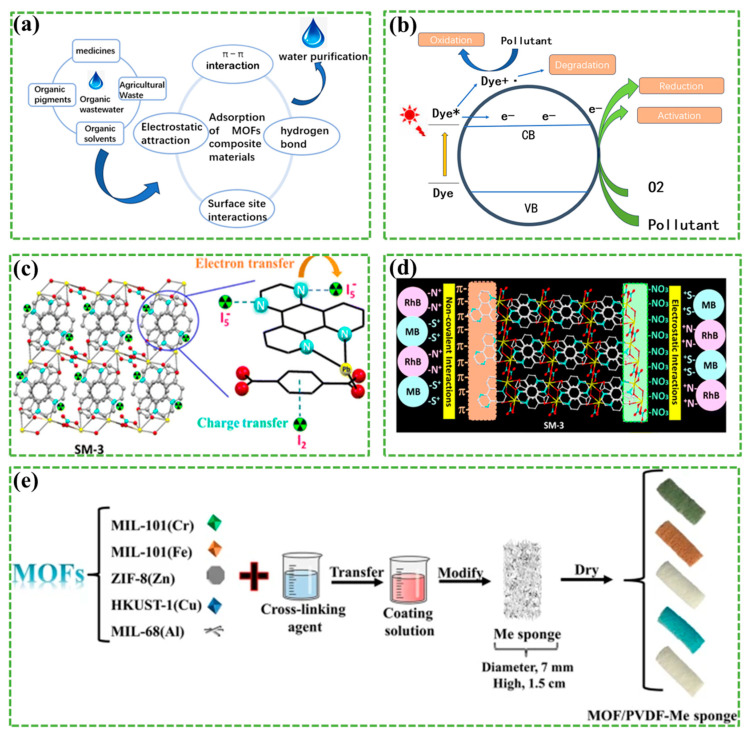
(**a**) Diagram of the MOF-based materials for purifying seawater. (**b**) The mechanism of MOF treatment of organic dyes (CB: conduction band; VB: valence band; Dye* and Dye+ are dye intermediates in different forms). (**c**) Possible mechanism of iodine adsorption by SM-3. Reprinted with permission from [99]. (**d**) Possible mechanism for cationic MB and RhB dye adsorption using MOF SM-3. Reprinted with permission from [99]. (**e**) Preparation of porous MOF/PVDF-sponge columns. Reprinted with permission from [100].

Furthermore, the practicality of MOFs hinges on the development of a simple and cost-effective synthesis method yielding high yields of MOF materials (Figure 6a). Various optimizations have been employed to produce numerous MOFs, such as ZIF-8, UIO-66, and MIL101, which have undergone extensive research for adsorption and detoxification applications [72,98].

### 4.1. Removal of Organic Dyes

Dyes represent a ubiquitous organic contaminant, prevalent in various manufactured goods, including paper, textiles, food, and pharmaceuticals, owing to their distinctive color and persistent nature as a water pollutant [101,102].

Numerous studies have been dedicated to finding effective solutions to prevent the discharge of industrial wastewater containing dyes into the environment. The presence of significant quantities of highly reactive and non-degradable chemicals in dyes released into the environment or tap water can disrupt ecosystems by harming plants and impacting animals. Textile wastewater remains a significant challenge to address [103,104]. Dyeing auxiliaries typically consist of inorganic salts, leading to wastewater containing a mixture of dye molecules and salts such as NaCl and Na_2_SO_4_. The presence of salt complicates the biodegradation of textile wastewater. Therefore, for resource recovery and environmental protection, the purification and removal of salt have become imperative [105,106].

To date, numerous studies have explored the application of MOFs in this context. With advancements in technology and research, the shortcomings of MOFs have also been addressed. Various types of MOFs have been developed, including modified MOFs, metal-organogels obtained by incorporating aerogel-hydrogels, and membrane-based MOFs. The mechanism of MOF treatment of organic dyes is illustrated in Figure 6b.

Saglam et al. investigated the efficacy of various MOF-based membranes, including modified MOFs, magnetic MOFs, aerogel hydrogels with MOFs, and metal-organogels (MOGs), in treating dye wastewater [107]. They found that modified MOFs exhibited the most effective removal of dye wastewater. In another case, Kamal et al. synthesized a 2D Pb(II) MOF (SM-3) using a mixed ligand method under solvothermal conditions [99]. SM-3 demonstrated excellent adsorption properties for iodine and dyes. The possible mechanism of SM-3 adsorption of iodine is illustrated in Figure 6c, while the potential mechanism for cationic MB and RhB dye adsorption using MOF SM-3 is depicted in Figure 6d.

Zhao et al. constructed thin film composite MOF membranes (TFCMMs) using spray deposition and post-stabilization technology [108]. Apart from exhibiting excellent stability, TFCMMs displayed a high proportion of MOF particles and a high-throughput dye retention rate. The sub-nm channels of MOFs can entrap dye molecules and facilitate water permeability. The ZIF-67-derived CoSX composite film, prepared by Sun et al. [109], exhibited exceptional permeability and remarkable dye molecule retention (>99.5%), with a salt retention rate of only 18.7%. The membrane demonstrated high durability, maintaining the separation process for over 100 h, and holds significant promise for dye desalination applications.

To mitigate the environmental impact of azo dye wastewater, a variety of treatment methods are employed, including chemical, physical, biological, and hybrid approaches [110]. MB, widely used in pharmaceuticals, textiles, and biological processing, poses significant environmental concerns due to its presence in industrial wastewater from dyeing cotton, silk, and wood [111]. MXene-MOF membranes demonstrated exceptional rejection capabilities for MB and RB, with rejection rates of 85.3% and 94.8%, respectively [112].

Apart from MB, various studies have highlighted the efficacy of MOFs in removing other dyes from wastewater. For instance, methyl orange (MO) has also been targeted for removal, with promising results. Researchers have developed DNA@ZIF-8/L-PDA films, achieving an impressive 97% inhibition rate for MO [113]. Additionally, Jiang et al. employed a straightforward method to synthesize a range of MOF materials with diverse structures, including MIL-101 (Cr), MIL-101 (Fe), ZIF-8 (Zn), HKUST-1 (Cu), and MIL-68 (Al) [100], as shown in Figure 6e. These MOFs were utilized to coat a sponge substrate, forming MOF/PVDF-sponge columns. This approach offers commercial potential owing to its simple one-step penetration, excellent adsorption properties, and ease of operation. Such research endeavors pave the way for exploring the adsorption of dyes from environmental water samples using functional porous sponges. Leveraging the high porosity and specific surface area of MOFs, these materials transcend mere filtration and exhibit remarkable photocatalytic activity, making them effective tools for degrading compounds that are typically resistant to breakdown, such as reactive dyes [114].

In fact, numerous studies have consistently shown that membranes incorporating MOFs demonstrate enhanced removal and decontamination of dyes compared to conventional substrates and membranes. The MOF film effectively eliminates a significant portion of the dye through electrostatic interactions on its surface. Despite the significant achievements in dye removal from wastewater, developing water-stable membranes with enhanced durability in aquatic environments remains a significant challenge.

### 4.2. Removal of Agricultural Pollutants

Agricultural pollutants encompass a broad range of substances, including fertilizers, pesticides, fungicides, herbicides, and growth regulators, as well as chemical compounds like organochlorines and excessive amounts of organophosphorus, nitrogen, potassium, and other soluble compounds. In contemporary agriculture, pesticides are extensively employed to safeguard fruits and vegetables from pests and diseases during cultivation and post-harvest [115].

Microplastics have emerged as persistent pollutants garnering global attention. In agricultural soils, the primary sources of microplastic pollution include the application of biosolids and compost, irrigation with wastewater, the use of mulch, polymer-based fertilizers and pesticides, and atmospheric deposition. Despite the presence of various pollutants in the soil environment, standardized detection and quantification technologies for microplastics remain lacking [116]. The widespread presence of pesticides in drinking water has led materials chemists to seek solutions for mitigating agrochemical contaminants in soil and water. Several porous nanostructures, including zeolite, silicates, porous carbon, and MXene, have shown promising results in this regard [117]. Adsorption can be considered a very effective method for cost-effective treatment of agricultural organic contaminants [118].

**Figure 7:**
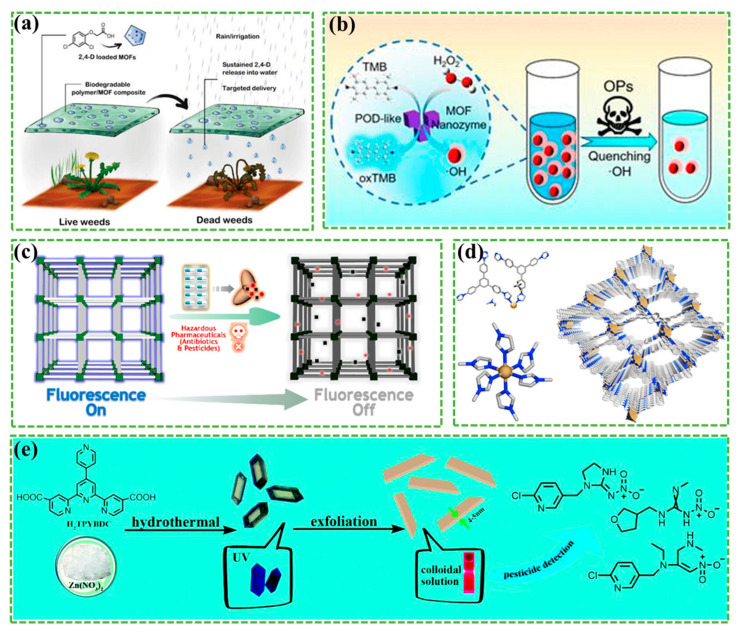
(**a**) MOF/polymer composites for targeted and sustainable pesticide delivery. Reprinted with permission from [121]. (**b**) MOF nanozyme-based colorimetric detection of OPs. Reprinted with permission from [127]. (**c**) iMOF-14C towards detection of hazardous antibiotics and pesticides, and (**d**) the structure of iMOF-14C. Reprinted with permission from [128]. (**e**) Two-dimensional Zn-LMOF nanosheets for pesticide sensing. Reprinted with permission from [129].

Due to their large specific surface area and significant porosity, MOF-based materials have emerged as excellent candidates for agricultural applications, particularly due to their high loading capacity and controlled release over extended periods. The use of MOFs for sustained release of agricultural chemicals was first reported in 2015, focusing on controlling the release of nitrogen and phosphate fertilizers [119]. In 2017, Yaghi et al. demonstrated the potential applications of two L-calcium lactate MOFs, MOF-1203 and MOF-1201, in controlled release fumigants, specifically cis-1,3-dichloropropene [120]. Despite promising results, the granular form of MOFs poses limitations in practical applications and raises concerns about potential environmental release. Reis et al. proposed a more sustainable approach to pesticide delivery using biodegradable MOF/polymer composites (Figure 7a) [121]. These composite membranes offer potential applications in controlled pesticide release during irrigation or rainfall, allowing targeted delivery of insecticides to weed-contact areas while minimizing contamination of non-target organisms and the surrounding environment.

Reda et al. investigated two different zeolite imidazole frameworks (ZIF-67 and ZIF-8) based on distinct metal ions (cobalt and zinc) for removing two common pesticides, propion and ethiophosphorus [122]. The surprising finding was the selectivity of the pesticides toward different metal ions. The maximum adsorption capacities of ZIF-8 and ZIF-67 were 366.7 and 261.1 mg g^−1^ for propion, respectively, and 279.3 and 210.8 mg g^−1^ for ethiophosphorus, respectively. Abdelhameed et al. synthesized a Cu-BTC@CA MOF membrane using in situ growth synthesis technology [123], which was employed to remove pesticides like methoate from wastewater. While the film exhibited a high absorption capacity, it showed some defects such as cracks.

More recently, Yu et al. developed porphyrin MOF-525 and MOF-545 (SMX) to eliminate sulfamethoxazole [124]. These MOFs possess strong adsorption capacity, large surface area, and reusability. Additionally, Mohamed et al. applied the new V_2_O_5_@Ch/CuTMA nanobiosorbent MOF [125], which exhibited improved removal efficiency for levofloxacin (LEVO) in water. Li and colleagues synthesized BiOX microrods from bismuth MOF [126]. These microrods, composed of BiOX photocatalysts, demonstrated excellent photocatalytic activity, adaptability, and reusability.

Despite few reports on the utilization of MOF derivatives and composites for the adsorption and catalytic removal of pollutants [130], Xiao et al. [127] proposed an MOF-mediated acetylcholinesterase (AChE)-free colorimetric strategy for direct detection of OP (Figure 7b). In the presence of organophosphorus, the dark blue color of oxidized 3,3′,5,5′-tetramethylbenzidine (TMB) lightens due to the quenching of hydroxyl radical (OH) by organophosphorus. This is facilitated by the decomposition of H_2_O_2_ catalyzed by Cu_4_Co_6_ZIF with excellent peroxidase (POD)-like activity. The developed colorimetric sensor exhibits favorable analytical properties and offers a promising strategy for detecting organophosphorus pesticides in real samples.

Dutta et al. discovered that a novel cationic 2D MOF, IMF-14C, demonstrates rapid and selective fluorescence shutdown recognition in water against harmful micropollutants, particularly nitro-functionalized antibiotics and pesticides [128]. The reaction mechanism is depicted in Figure 7c, and the structure of iMOF-14C is illustrated in Figure 7d. Furthermore, iMOF-14C exhibited high selectivity and sensitivity in recognizing specific pesticides such as CHPS and nitrofen. Overall, iMOF-14C was observed to selectively detect electron-deficient micropollutants over its electron-rich congeners, forming the basis for its conversion into iMOF-14C@PVDF, a typical MOF@polymer composite membrane. In another study, Reda et al. incorporated MOF into functionalized cotton, effectively removing pesticides and dyes with the developed hybrids [131]. The maximum adsorption capacity for pesticides and pigments of the cotton hybrid surpassed that of other pollutants, with the highest adsorption observed for cotton and Zr-MOF@OX.

In addition, Tengles et al. synthesized a novel MOF using a single layer of Fe_3_O_4_-ZIF-8 [132]. This synthesized MOF was employed as an absorbent to eliminate fipronil from cucumber samples, showcasing a large surface area and excellent adsorption capacity. Moreover, Fe_3_O_4_-ZIF-8 consists of numerous similar pore structures, which aid in the removal of fipronil from vegetable samples.

Su et al. synthesized a new luminescent metal–organic framework (LMOF) Zn-LMOF, stripped into ultra-thin 2D nanosheets (4–5 nm) (Figure 7e) [129], by disrupting the weak interaction between the coordination layers (forming a luminescent colloidal sensor) and the π-π interaction between the conjugated aromatic rings within the layers. It can sensitively detect pesticides such as imidacloprid, endinidamine, and furosemide, exhibiting fluorescence quenching effects with a very low detection limit (LOD). Using imidacloprid as a typical case, the LOD value for simulated agricultural environment samples is 0.562 μM, with a recovery rate in the range of 94–115%, indicating that the prepared 2D Zn-MOF nanoparticle colloidal sensor (Zn-LMOF probe) is the most promising candidate for sensing chemical pesticides.

### 4.3. Removal of Oil Contaminants

**Figure 8:**
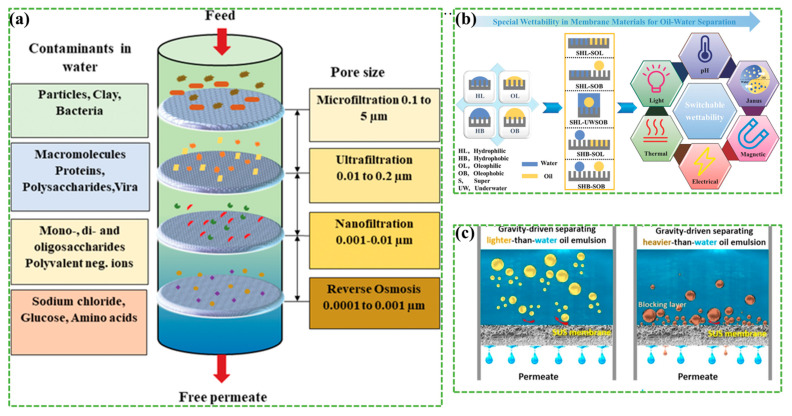
(**a**) Schematic illustration of pressure-driven membrane filtration processes. Reprinted with permission from [135]. (**b**) MOF membrane materials for oil/water separation. Reprinted with permission from [139]. (**c**) Polymeric membranes for oil/water separation behaviors. Reprinted with permission from [140].

With the advancement of global industrialization, various sources such as kitchens, petrochemical industries, and accidental spills contribute to the generation of oil/water mixtures, necessitating urgent action to address the discharge of oily wastewater and oil spills. Consequently, the treatment of oily wastewater has become a matter of significant importance, drawing widespread attention. Conventional treatment methods, including gravity separation [133], chemical reaction, physical adsorption, centrifugation, and biodegradation, suffer from drawbacks such as high energy consumption, low separation efficiency, and high costs [134]. In contrast, membrane separation technology has emerged as an efficient solution in both gravity and pressure-driven membrane processes. These membranes can effectively remove different types and sizes of suspended particles, as depicted in Figure 8a [135]. The performance of these membranes depends on various factors including membrane thickness, permeability, filtration time, feed concentration, and transmembrane pressure [136]. Microfiltration is particularly effective in removing suspended micron and nanoscale solid particles, with pore sizes typically ranging from 0.05 to 10 µm.

Due to their excellent properties, MOF membranes show promise for the removal of oily wastewater. MOF films exhibit varying degrees of affinity to oil-containing pollutants on their surfaces. Goh and Ismail [137] investigated the potential synergies between rich porosity and ultra-wettability to enhance the superhydrophobicity and superlipophilicity of membranes, thereby enhancing their effectiveness in treating oily wastewater. According to the Cassie–Baxter model, enhancing the roughness of a surface’s micro-nano structure can improve surface wettability, resulting in a superhydrophobic/superlipophilic material. This material, known for its selective separation ability in terms of oil absorption and ease of manufacture, has garnered significant interest [138]. Figure 8b summarizes some membrane materials with special wettability for oil/water separation [139].

Xiu et al. prepared polylactic acid(PLA)/ZIF-8 membranes by incorporating ZIF-8 NPs into PLA to fabricate composite electrospinning membranes for oil/water separation [141]. Increasing the ZIF-8 content enhanced the film’s roughness and reduced the diameter of the blended fibers. This adjustment significantly improved the lubricity of the oil and enhanced the separation efficiency. The wood/MIL100 (Fe) membranes for oil/water separation achieved a remarkable separation rate of up to 99.9% due to factors such as hydrophilicity and pore size optimization [142]. Another effective membrane for oil/water separation is polyetherimide(PEI)/polyvinyl pyrrolidone(PVP)/MOF-71, which boasts a retention rate of 85.4% [76]. These membranes utilize hydrophobic, hydrogen-bonding, and π-π interactions for efficient purification. A simplified binder-free strategy for manufacturing robust superhydrophobic polymeric membranes for oil/water separation is shown in Figure 8c [140]. The MOF-based membranes for oil/water separation followed this principle.

A kind of HKUST-1 MOF-coated foam copper for oil/water separation was prepared by a simple template sacrifice method. The prepared copper foam had good superhydrophobicity under wide pH values and salt-water conditions. Its oil absorption capacity for all selected oils was more than 150%, and the oil/water separation efficiency of the foam for various oil/water mixtures was more than 96%. In addition, although the wettability decreased to a hydrophobic state after 30 mechanical wear cycles, the separation efficiency of the foam remained above 97%, showing good repeatability. The results showed that the unique 3D structure of foam copper contributes to excellent mechanical wear durability [143].

He et al. prepared a novel superhydrophilic and underwater superoleophobic material (MOF-5/PDA/SSM) with high throughput, excellent oil/water separation performance, and durability. Stainless steel mesh (SSM) was modified with MOF-5 crystals and polydopamine (PDA). The introduction of MOF-5 can improve the hydrophilicity of SSM while achieving high water flux. The MOF-5 particles on the mesh were cubic hexahedral crystals with a uniform size and diameter, which can trap water in the rough microstructure and form a strong hydration barrier layer or water solid composite interface on its surface, hindering the penetration of oil. The grid also showed significant water flux and separation performance of various oil/water mixtures. In particular, it was found that the successfully prepared materials exhibited significant stability in various physical and chemical extreme environments, including maintaining UWOCA > 150 in acidic/alkaline media, performing well after continuous separation for 120 min, and exhibiting high resistance to oil intrusion pressure [144].

Habibi et al. modified flexible superhydrophobic/superoleophilic polyurethane (PU) sponges by applying Cu(BDC)MOFs as flame retardants and nano graphite sheets (NGP) as superhydrophobic agents [145]. These materials were attached to the sponge skeleton using poly (styrene butyl acrylate). The MOF-based sponge exhibited high thermal stability, mechanical stability, and chemical stability. In addition, the composite material revealed excellent oil/water separation efficiency. The oil adsorption capacity at a static state and oil separation efficiency at a continuous oil/water separation process for PU-Cu (BDC)-NGPs sponge were 43.5–132 g/g and 96%, respectively.

The functions and properties of MOF-based membranes are crucial for the efficiency of oil/water separation in practical applications. For instance, Cao et al. synthesized a MOF, polyacrylic acid (PAA) modified UiO-66-NH_2_ (UiO-66-NH_2_@PAA), and used it to fabricate membranes through a vacuum-assisted self-assembly process [146]. Due to the high water-absorption capacity of the MOF, rich hydrophilicity, presence of negatively charged carboxyl groups, and high surface roughness, the film exhibited high hydrophilicity and underwater superoleophobic properties, leading to better anti-fouling performance. The MOF film showed high separation efficiency for oil/water emulsions (rejection rate > 99.9%). Under optimal conditions, the pure water flux was 2330 L/m^2^ h bar, and the flux recovery rate (FRR) remained above 80% after three cycles of oil/water emulsion separation, demonstrating the great potential of MOF membranes in oil/water separation.

**Figure 9:**
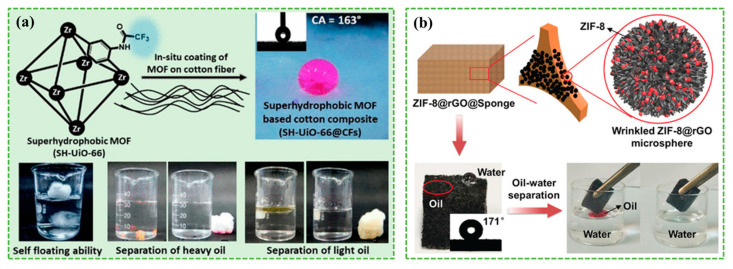
(**a**) Synthesis of SH-UiO-66@CFs for oil/water separation. Reprinted with permission from [147]. (**b**) ZIF-8@rGO microspheres for oil/water separation. Reprinted with permission from [148].

Dalapati et al. synthesized a superhydrophobic MOF with UiO-66 (SH UiO-66) topological structure, as shown in Figure 9a, which revealed a new fluorinated dicarboxylate linker to selectively absorb oil from water. The synthesized porous MOF with high WCA (SBET = 873 m^2^ g^−1^) showed good application prospects in oil/water separation [147]. Further coating of superhydrophobic SH-UiO-66 onto cotton fibers (CFs) for the formation of SH-UiO-66@CFs was carried out, which could be utilized to separate oil form the oil/water mixtures through gravity-guided active filtration. In another case, Gu et al. embedded MOF nanomaterials into GO NPs to create ZIF-8@rGO@ sponge films through self-assembly at high temperatures (Figure 9b) [148], resulting in films with remarkably high separation efficiency (>98%) and adsorption selectivity for oily wastewater. These films exhibited a synergistic effect in oil/water separation. Moreover, they demonstrated excellent recyclability, maintaining their effectiveness even after 100 cycles.

Much of the research in this field focuses on isolating individual components, often conducted on a laboratory scale. Designing and manufacturing MOF membranes capable of efficiently removing contaminants from real wastewater in treatment plants to purify the water from microcontaminants represents a significant challenge and an area worthy of further study.

To make the above introduction clearer, we provide a table (Table 1) to summarize the MOF-based materials for the treatment of organic effluents.

## 5. Conclusions and Outlooks

MOF-based materials have been widely utilized in organic wastewater treatment due to their high specific surface area, controllable pore structure, and strong adsorption and catalytic properties. In this review, we comprehensively examine recent advances in the application of MOF-based materials for organic wastewater treatment. We present various synthesis methods, including traditional solvothermal, non-solvothermal, and novel electrochemical and microwave methods. Additionally, we explore the functionalization of MOF-based materials and their impact on adsorption and photocatalytic degradation performance in organic wastewater treatment. Finally, this review provides a detailed discussion on the application of MOF-based materials in organic wastewater treatment. We anticipate that further research in this field will reveal additional applications for MOF-based materials and their composites, contributing to significant scientific advancements.

MOF-based materials have demonstrated significant potential in wastewater purification by effectively removing various inorganic and organic contaminants. Numerous important studies have been conducted in this area, laying the groundwork for future advancements. Here, we present several key perspectives on the future development of MOF materials for environmental science. Firstly, there is a need for both structural and functional tailoring of MOF-based materials. This involves adjusting the porous structure and specific surface area of MOF materials to enhance their adsorption capacity for pollutants. Secondly, hybridizing MOFs with photoactive nanomaterials can lead to the creation of multifunctional MOF materials with high adsorption ability and photocatalytic functions. These photo-responsive materials could effectively degrade various organic contaminants through photocatalysis. Thirdly, research should focus on synthesizing MOF-based materials for the adsorption and degradation of volatile organic compounds (VOCs) in the air. By binding MOFs with photoactive nanomaterials such as nanozymes, synergistic effects can enhance the removal performance of MOFs regarding VOCs. Fourthly, MOFs can be combined with nanozymes to form MOF/nanozyme composites, which can serve as colorimetric sensors for the rapid and effective determination of heavy metal ions, anions, and other organic contaminants. Lastly, there is a need to strengthen performance evaluation and application research of MOF materials in real environmental treatment scenarios. Furthermore, efforts should be made to promote the large-scale preparation and industrial application of MOF materials to achieve widespread utilization in environmental governance.

## Figures and Tables

**Table 1:** Summary of MOF-based materials for treatment of organic effluents.

MOF-Based Material	Pollutants Removed	Preparation Method	Advantages	Ref.
SM-3	Iodine and dyes	Solvothermal method	High selectivity	[99]
MIL-101 (Cr), MIL-101(Fe)	Methyl orange dye	Infiltration method	Excellent adsorption capacity, simple one-step infiltration, excellent adsorption properties and simple operation	[100]
ZIF-L, UiO-66-NH_2_	Dye	Spray deposition and post-stabilization	High water permeance and excellent dye rejection	[108]
CoSx composite membrane ZIF-67-derived CoSx	Dye Desalination of dyes	Solvothermal method In situ sulfurization	Outstanding permeance and excellent rejection	[109]
xO_y_=Ag_2_O and ZnO	MB and MO azo dyes	Sol–gel method	Definite durability	[110]
iMOF-14C	Antibiotics and pesticides	Solvothermal method	High selectivity and low detection limit	[128]
Cu-BTC@ CA	Dimethoate pesticide	In situ synthesis	High adsorbability, large adsorption capacity and good recoverability	[123]
MOF-525 and MOF-545	Sulfamethoxazole	Solvothermal method	Great adsorption capacity and good reusability	[124]
V_2_O_5_@Ch/Cu-TMA	Levofloxacin drug and Cr(Vl)	Solvothermal method	High efficiency	[125]
NH2-UiO-101-Zr@Ox-cotton	Dye	Non-aqueous method	Large adsorption capacity and good recyclability	[131]
M-ZIF-8@ZIF-67	Fipronil and its metabolites	Solvothermal method	High adsorption capacity	[132]
PVDF membranes	Oil	Delayed phase inversion	Good stability and excellent recyclability	[140]
PLA/ZIF-8	Dye wastewater	Solvothermal method	Increased oil wettability and significantly improved mechanical property	[141]
HKUST-1 MOFs	Oil	Solvothermal method	High separation efficiency and good repeatability	[143]
MOF-5/PDA/SSM	Oil	Solvothermal method	Outstanding separation efficiency, excellent recyclability and high water flux	[144]
Cu(BDC)MOF	Oil	Solvothermal method	Excellent stability, exhibited good compressibility and reusability	[145]
SH-UiO-66	Heavy and light oils	Solvothermal method	High water resistance, high oil absorption capacity and good reversibility	[147]
Ti3C2-Mxene	Oil	Solvothermal method	High hydrophobicity, abundant porosity and sufficient mechanical strength	[149]
NH2-MIL-125 (Ti)	Pesticide	Solvothermal method	High removal rate	[149]

## Data Availability

Data can be requested from the authors.
